# Antiviral Activity of a Small Molecule Deubiquitinase Inhibitor Occurs via Induction of the Unfolded Protein Response

**DOI:** 10.1371/journal.ppat.1002783

**Published:** 2012-07-05

**Authors:** Jeffrey W. Perry, Mohammad Ahmed, Kyeong-Ok Chang, Nicholas J. Donato, Hollis D. Showalter, Christiane E. Wobus

**Affiliations:** 1 Department of Microbiology and Immunology, University of Michigan Medical School, Ann Arbor, Michigan, United States of America; 2 Department of Diagnostic Medicine and Pathobiology, College of Veterinary Medicine, Kansas State University, Manhattan, Kansas, United States of America; 3 Department of Internal Medicine, University of Michigan Medical School, Ann Arbor, Michigan, United States of America; 4 Vahlteich Medicinal Chemistry Core, Department of Medicinal Chemistry, College of Pharmacy, University of Michigan, Ann Arbor, Michigan, United States of America; University of North Carolina at Chapel Hill, United States of America

## Abstract

Ubiquitin (Ub) is a vital regulatory component in various cellular processes, including cellular responses to viral infection. As obligate intracellular pathogens, viruses have the capacity to manipulate the ubiquitin (Ub) cycle to their advantage by encoding Ub-modifying proteins including deubiquitinases (DUBs). However, how cellular DUBs modulate specific viral infections, such as norovirus, is poorly understood. To examine the role of DUBs during norovirus infection, we used WP1130, a small molecule inhibitor of a subset of cellular DUBs. Replication of murine norovirus in murine macrophages and the human norovirus Norwalk virus in a replicon system were significantly inhibited by WP1130. Chemical proteomics identified the cellular DUB USP14 as a target of WP1130 in murine macrophages, and pharmacologic inhibition or siRNA-mediated knockdown of USP14 inhibited murine norovirus infection. USP14 is a proteasome-associated DUB that also binds to inositol-requiring enzyme 1 (IRE1), a critical mediator of the unfolded protein response (UPR). WP1130 treatment of murine macrophages did not alter proteasome activity but activated the X-box binding protein-1 (XBP-1) through an IRE1-dependent mechanism. In addition, WP1130 treatment or induction of the UPR also reduced infection of other RNA viruses including encephalomyocarditis virus, Sindbis virus, and La Crosse virus but not vesicular stomatitis virus. Pharmacologic inhibition of the IRE1 endonuclease activity partially rescued the antiviral effect of WP1130. Taken together, our studies support a model whereby induction of the UPR through cellular DUB inhibition blocks specific viral infections, and suggest that cellular DUBs and the UPR represent novel targets for future development of broad spectrum antiviral therapies.

## Author Summary

Deubiquitinases (DUBs) are enzymes, which are implicated in many cellular processes but their functions during virus infection are not well understood. We used WP1130, a small molecule inhibitor of a subset of DUBs, as a probe to unravel the functions of DUBs during norovirus infections. We identified USP14 as a cellular DUB target of WP1130 that is required for optimal norovirus infection. Furthermore, we demonstrated that chemical induction of the unfolded protein response can significantly inhibit viral progeny production of several RNA viruses, including noroviruses. These results suggest that chemical inhibition of cellular DUBs and/or modulation of the unfolded protein response could represent novel targets for therapy against a variety of viral pathogens.

## Introduction

Noroviruses are small non-enveloped viruses with positive-strand RNA genomes [1]. Human Norovirus (HuNoV) is the major cause of sporadic and epidemic non-bacterial gastroenteritis worldwide in people of all ages [2], [3]. Typically these infections result in high morbidity and economic costs but occasionally cause mortality [4], [5], [6]. However, no directed antiviral treatments or vaccination strategies are currently available to prevent or control norovirus outbreaks. This is in part due to the inability to reproducibly culture HuNoV in the laboratory, which has seriously hampered studies of this pathogen [7], [8], [9]. Recently, a replicon system was developed by stably expressing a plasmid containing the prototypic norovirus strain, Norwalk virus, and an antibiotic resistant cassette enabling limited studies on the replication requirements of HuNoV [10], [11], [12]. In addition, the discovery of murine norovirus 1 (MNV-1) and identification of murine macrophages and dendritic cells as permissive cell types led to the development of the first norovirus cell culture system [13], [14], [15]. MNV shares many biological and molecular properties with HuNoV [15]. Like its human counterparts, MNV is an enteric virus that is infectious after oral inoculation, replicates in the intestine and is shed in the stool, resulting in fecal-oral transmission [15]. MNV also shares the typical genomic organization, biophysical properties of the viral capsid, and molecular mechanisms of translation initiation with HuNoV [15], [16], [17]. Therefore, research using MNV is increasingly uncovering principles of norovirus biology.

The ubiquitin (Ub) cycle is required for many cellular processes, including proteasomal degradation [18] and the unfolded protein response (UPR) (*e.g.* [19], [20], [21]), a cellular process whereby cells respond to the accumulation of unfolded proteins in the endoplasmic reticulum (ER) and other environmental stresses [22]. Ub-conjugating and Ub-deconjugating processes are precisely and tightly regulated, and dysregulation can lead to disease (*e.g.* [23], [24], [25], [26]). Ub is a small 76 amino acid protein that can be covalently linked to cellular proteins in a post-translational manner through a series of Ub-modifying enzymes [27]. Removal of Ub by deubiquitinases (DUBs) is a critical step to counterbalance Ub conjugation. DUBs are a group of cysteine proteases that process poly-Ub during protein translation, recycle partially catalyzed Ub intermediates, remove Ub from target proteins, and process free polymeric Ub chains cleaved from target proteins [28], [29]. Based on common structural features, DUBs are divided into five families, including the ubiquitin C-terminal hydrolases (UCH) and the ubiquitin-specific proteases (USPs) [30]. USPs are the largest and most diverse DUB family and target proteins with Ub modifications. In addition to the well-characterized roles of DUBs in proteasomal degradation [31], DUBs have been implicated in regulating other universal cellular processes such as the UPR [32]. The sensors inositol-requiring enzyme 1 (IRE1), PKR (double-stranded-RNA-dependent protein kinase)-like ER Kinase (PERK), and activating transcription factor 6 (ATF6) initiate the three arms of the UPR, which collectively upregulate ER chaperone expression, increase ER-associated degradation (ERAD), and attenuate protein translation to reduce the amount of misfolded proteins in the ER [22]. A recent study demonstrated that USP14 interacts with the cytoplasmic region of IRE1 to inhibit ERAD under non-stress conditions [32]. While details of the USP14-IRE1 regulation remain to be determined, these studies suggest a critical role for ubiquitin and DUBs in the UPR.

As obligate intracellular pathogens, many viruses manipulate the Ub cycle to their advantage by hijacking cellular Ub-modifying enzymes, including DUBs, or by encoding proteases and isopeptidases that recognize Ub-modified proteins [28]. However, how cellular DUBs function in modifying viral infections is poorly understood. One recent study showed that the cellular DUB USP11 restricts influenza A virus replication [33]. The monoubiquitinated nucleoprotein associates with the ribonucleoprotein complex during viral replication. USP11 can cleave monoubiquitin from the nucleoprotein, inhibiting colocalization of the nucleoprotein in ribonucleoprotein complexes and significantly inhibiting viral replication. This suggests that DUBs may function as cell-intrinsic restriction factors during virus infections, but whether DUBs also promote virus infections is unknown. Furthermore, the role of DUBs during norovirus infection has not previously been addressed.

To examine the role of DUBs during norovirus infection, we used a small molecule, WP1130, which inhibits a subset of cellular DUBs [34]. WP1130 is a cell permeable inhibitor of DUBs that induces the accumulation of ubiquitinated proteins in multiple cell lines including the MNV-permissive murine macrophage line RAW 264.7 [34], [35]. In Z138 mantle cell lymphoma cells, WP1130 inhibits USP9x, USP5, USP14, UCH37, UCH-L1, and potentially other DUBs [34]. In addition to its anti-cancer activity [34], [36], [37], WP1130 has anti-bacterial effects since treatment enhances killing of *Listeria monocytogenes* in murine macrophages [35]. Herein, we show that WP1130 also significantly inhibited MNV-1 infection in murine macrophages and genomic replication of Norwalk virus in the replicon system. USP14, a proteasome-associated DUB [38], was subsequently identified as a target of WP1130 in murine macrophages. Inhibition of USP14 activity reduced MNV-1 infection but WP1130 did not inhibit proteasome activity. Instead, WP1130 treatment activated the UPR. Pharmacologic activation of the UPR with thapsigargin, an inhibitor of the sarco/endoplasmic reticulum calcium ATPase [39], also significantly inhibited MNV-1 infection. This effect was not limited to noroviruses or murine macrophages. A similar inhibition of viral infection by WP1130 was demonstrated in African green monkey kidney (Vero) and human neuroblastoma (Be2-c) cells with several RNA viruses including, encephalomyocarditis virus (EMCV), Sindbis virus, and La Crosse virus but not vesicular stomatitis virus (VSV). In all cases, the antiviral activity of WP1130 was partially reversed by inhibition of IRE1 endonuclease activity. In addition, WP1130 also significantly decreased MNV-1 infection near the injection site in the jejunum/duodenum of mice. Taken together, our results suggest that WP1130 restricts viral replication in part through the IRE1-dependent UPR, which is activated upon inhibition of DUBs. Thus, DUB inhibitors and UPR activators could provide a novel approach in antiviral therapy.

## Results

### The small molecule DUB inhibitor WP1130 inhibits MNV-1 replication

**Figure 1:**
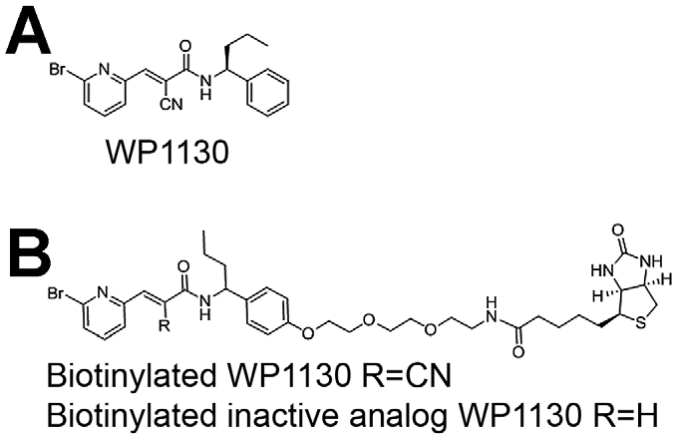
Chemical structures of WP1130 and its derivatives used herein. (A) WP1130, (B) biotinylated WP1130 and an inactive analog.

**Figure 2:**
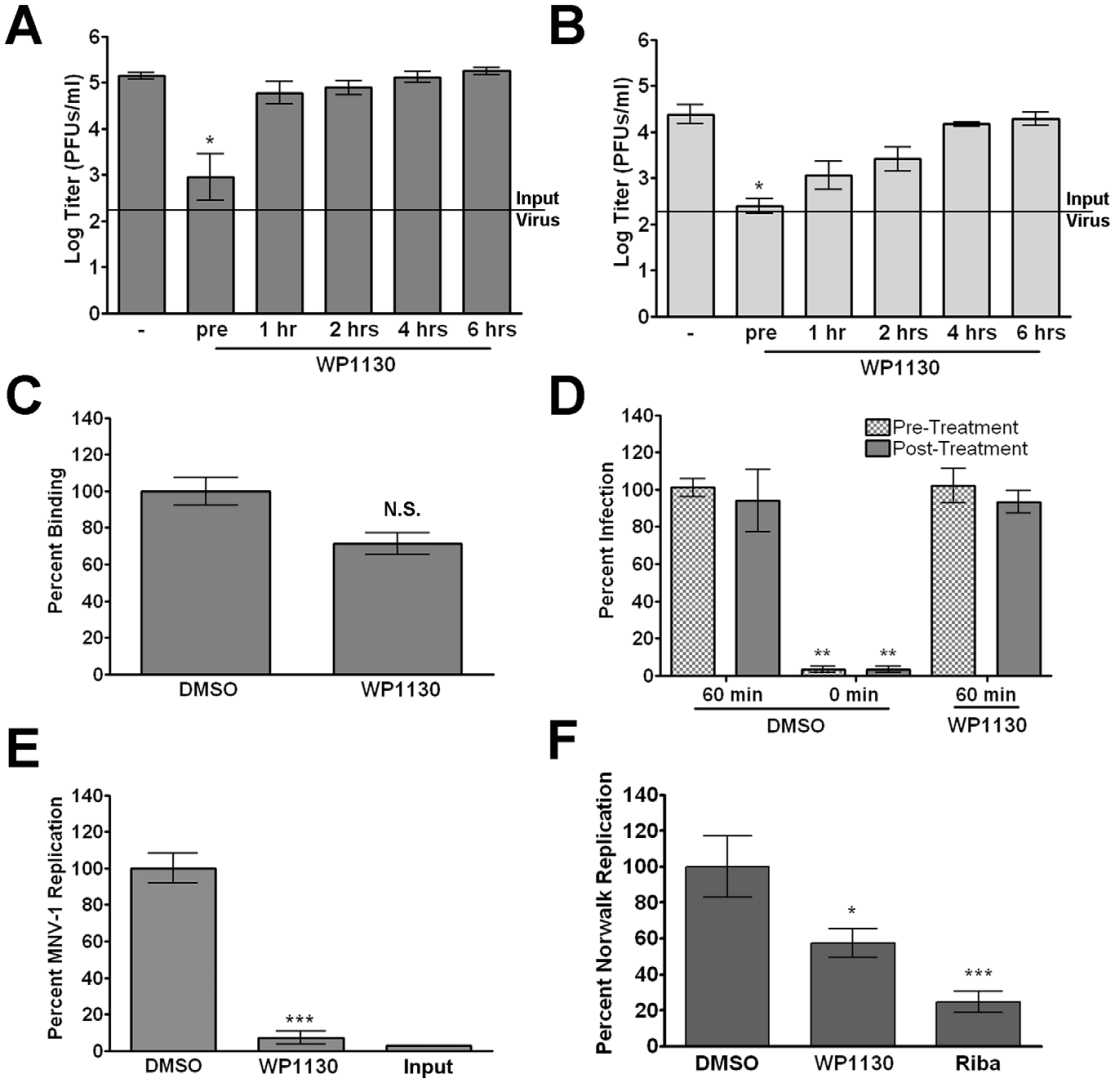
WP1130 treatment inhibits norovirus replication. (A, B) WP1130 treatment inhibits MNV-1 infection in (A) RAW 264.7 (RAW) cells or (B) bone marrow-derived macrophages (BMDMs). Cells were infected with MNV-1 (MOI 5) in the presence of 5 µM WP1130 or DMSO (−). Cells were incubated with WP1130 30 min prior to infection (pre) or at the indicated times post-infection. Virus titers were determined by plague assay 8 hours (RAW) or 12 hours (BMDMs) post-infection. (C) MNV-1 attachment to murine macrophages is not significantly altered by WP1130 treatment. MNV-1 (MOI 5) was incubated for 1 hour on ice with RAW cells treated with 5 µM WP1130 or DMSO. Virus attachment was quantified by qRT-PCR. (D) WP1130 does not inhibit MNV-1 entry. RAW cells were infected with neutral red-containing MNV-1 (MOI 0.001) for 60 min at room temperature and then illuminated with white light. Cells were either treated prior to infection (pre-treatment) or treated for 90 minutes after infection (post-treatment) with 5 µM WP1130 or DMSO. To show the dynamic range of the assay, cells treated with DMSO were also illuminated with white light at the same time as infection was initiated (0 min). (E) WP1130 treatment inhibits MNV-1 replication. MNV-1 genomic RNA was transfected into RAW cells and quantified either 12 hours (Input) or 24 hours later using qRT-PCR. Cell were treated with DMSO or 5 µM WP1130 for the final twelve hours. MNV-1 genome copy number was normalized to DMSO-treated samples. (F) WP1130 treatment inhibits Norwalk virus replication. Norwalk virus replicon-bearing HG23 cells were treated with DMSO, 5 µM WP1130, or 100 µg/ml Ribavirin for 24 hours and Norwalk virus genomes quantitated by qRT-PCR. Norwalk virus genome copy number was normalized to DMSO-treated samples. In all cases, data from at least three independent experiments with two experimental replicates per condition are presented as means +/− S.E.M. *P<0.05, **P<0.01 and *** *P*<0.001, N.S. non-significant.

The role of cellular DUBs during norovirus infection has not been investigated. Towards that end, we used WP1130, a small molecule that inhibits a subset of DUBs [34] (Fig. 1). Murine macrophages were treated with 5 µM WP1130 for 30 minutes prior to MNV-1 infection (strain MNV-1.CW3), and viral titers were determined by plaque assay (Fig. 2A, B). Pre-treatment with WP1130 significantly reduced viral titers in both RAW 264.7 (RAW) cells, a murine macrophage cell line (Fig. 2A), and primary bone marrow-derived macrophages (BMDMs) (Fig. 2B). Interestingly, the antiviral effect of WP1130 was only observed during the early stages of infection. Addition of the compound post-infection (1 hour after infection for RAW cells or 4 hours after infection for BMDMs) ablated WP1130 antiviral activity (Fig. 2A, B). Under the same conditions, the compound's effect on mitochondrial dehydrogenase activity, an indicator for cell viability, was not significantly different from the DMSO control as measured by WST-1 reagent (Roche) (Fig. S1). Overall, these results suggested that WP1130 inhibits MNV-1 infection of murine macrophages, but only when added to cells before or early during infection.

These results raised the possibility that WP1130 was effective at an early step in the MNV-1 life cycle. To determine the effect of WP1130 treatment on viral attachment, the amount of viral particles bound to cells was measured using a qRT-PCR attachment assay previously described by our laboratory [40] (Fig. 2C). RAW cells were incubated with vehicle control (DMSO) or 5 µM WP1130 prior to infection, infected with MNV-1 on ice, washed, and cell-attached viral genomes were quantitated (Fig. 2C). While the genome levels on WP1130-treated cells were slightly decreased compared to DMSO-treated cells, this difference was not statistically significant, suggesting that MNV-1 attachment was not affected by WP1130 treatment.

We next examined the effect of WP1130 treatment on viral entry (*i.e.* attachment, internalization, and uncoating) using the neutral red assay previously adapted for use with MNV in our laboratory [41] (Fig. 2D). Neutral red, a photo-activated chemical, is passively incorporated into viral particles, which when exposed to white light cross-links the viral genome to the protein coat and renders the virus non-infectious [42]. This assay enables examination of MNV entry in the presence of inhibitor without impacting later stages of the viral life cycle. RAW cells were treated with WP1130 or DMSO prior to or after infection with neutral red-containing MNV-1 (Fig. 2D). To show the dynamic range of the assay, RAW cells treated with DMSO were illuminated at the same time as viral infection was initiated (Fig. 2D, DMSO 0 min) or after 60 minutes (Fig. 2D, DMSO 60 min) when MNV-1 was previously shown to become insensitive to light inhibition [41]. The number of infectious events was normalized to the DMSO control at 60 minutes prior to illumination (Fig. 2D, DMSO 60 min). WP1130 treatment did not alter viral infectivity when RAW cells were treated with WP1130 either prior to infection (Fig. 2D, WP1130 pre-treatment) or after 60 minutes of infection (Fig. 2D, WP1130 post-treatment). Interestingly, a reduction in plaque size was observed for WP1130-treated samples, although this was not statistically different from the DMSO control samples. This suggested that WP1130 may have inhibited viral infection in the early stages of plaque development, but not for the entire length of the experiment. Taken together, these findings suggested that WP1130 does not inhibit attachment, internalization, or uncoating of MNV-1 in RAW cells.

A critical next step in the viral life cycle following entry is replication. Thus, to determine whether WP1130 treatment inhibited viral replication, MNV-1 genomes were isolated from infected cell lysates and transfected into RAW cells (Fig. 2E). MNV-1 genomes were quantified by qRT-PCR [40] either after transfection for 12 hours but prior to treatment (Fig. 2E, input), or after an additional 12 hour treatment with WP1130 or DMSO (Fig. 2E). WP1130 treatment significantly reduced the number of MNV-1 genomes compared to DMSO-treated cells, demonstrating that WP1130 inhibited viral replication.

It is currently not possible to follow the full infectious cycle of HuNoV in a laboratory setting [7], [8]. However, the Norwalk virus replicon system measures Norwalk virus genomic replication [10]. Thus, we next determined whether WP1130 treatment also inhibited replication of Norwalk virus (Fig. 2F). Replicon-bearing hepatoma HG23 cells were grown in the presence of WP1130 or DMSO for 24 hours, and Norwalk virus genomes quantitated using qRT-PCR as previously described [10] (Fig. 2F). The number of Norwalk virus genomes was reduced approximately 50% upon treatment with WP1130 (Fig. 2F). As a positive control, HG23 cells were treated with ribavirin, a nucleoside analog that inhibits Norwalk virus replication [11]. Similar to previous findings [11], ribavirin reduced replication to approximately 20% of the DMSO-treated cells. These results demonstrated that WP1130 significantly inhibits Norwalk virus replication.

Taken together, our findings demonstrated that WP1130 is an effective inhibitor of MNV-1 and Norwalk virus replication but did not block the earlier stages of MNV-1 infection, namely attachment and entry. Since WP1130 is a known inhibitor of a subset of DUBs [34], these results suggested that all or some of the WP1130-responsive cellular DUBs are important for optimal norovirus replication.

### WP1130 treatment inhibits the cellular deubiquitinase USP14

**Figure 3:**
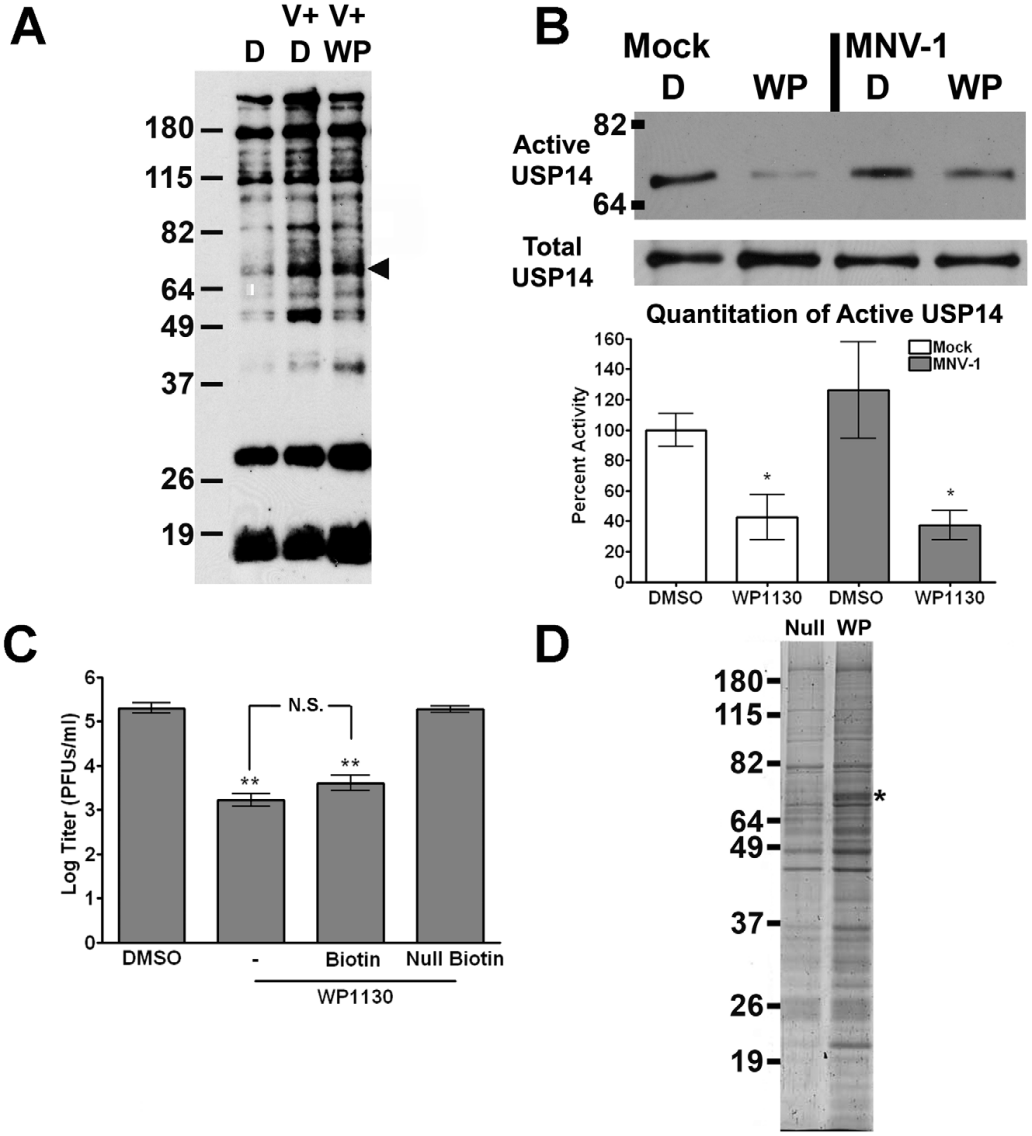
WP1130 inhibits the host deubiquitinase USP14 in murine macrophages. (A) WP1130 treatment inhibits the activity of multiple DUBs in murine macrophages. RAW cells were treated with DMSO (D, V+D) or 5 µM WP1130 (V+WP) for 30 minutes prior to infection. Cells were then infected with MNV-1 (V+D, V+WP) or mock lysate (D), washed, and incubated for an additional hour. Cell lysates were incubated with a non-hydrolysable ubiquitin conjugated to an HA tag (HA-UbVS) before separation by SDS-PAGE and immunoblotting with an anti-HA antibody. The experiment was performed three times and a representative blot is shown. A band of the anticipated molecular weight for USP14 is indicated by the arrow head. (B) WP1130 treatment inhibits USP14 activity. RAW cells were treated with DMSO (D) or 5 µM WP1130 (WP) and then infected with MNV-1 (MOI 5) or mock lysate, washed, and incubated for an additional hour. Cell lysates were labeled with HA-UbVS and immunoprecipitated using an anti-HA antibody. Proteins were separated by SDS-PAGE and immunoblots performed using an anti-USP14 antibody. A representative blot is shown (top, Active USP14). Densitometry was performed on four independent experiments, quantitated, and normalized to the mock- and DMSO-treated sample (bottom, Quantitation of Active USP14). As a control, immunoblots were performed for total USP14 levels in cell lysates prior to DUB labeling (middle, Total USP14). (C) Biotinylated WP1130 inhibits MNV-1 infection in RAW cells. Cells were treated with DMSO or 5 µM of WP1130 (WP1130), biotinylated WP1130 (Biotin), inactive biotinylated WP1130 analog (Null Biotin) prior to MNV-1 infection (MOI 5). Viral titers were determined by plaque assay 8 hours post-infection. Data from three independent experiments with two experimental replicates per condition are presented as means +/− S.E.M. **P<0.01, N.S. non-significant. (D) Biotinylated WP1130 interacts with USP14. RAW cells were treated with 5 µM of biotinylated WP1130 (WP) or the inactive biotinylated WP1130 analog (Null), lysed, and lysates incubated with streptavidin beads. Precipitated proteins were separated by SDS-PAGE and visualized with Ruby Red protein stain. Peptides corresponding to USP14 were recovered from the band indicated by the asterisk (*) by mass spectrometry.

We next sought to identify DUBs that may mediate the antiviral activity in macrophages observed during WP1130 treatment. Towards that end, we employed two independent labeling strategies; first, an activity-based DUB labeling assay, and second, a biotinylated WP1130 to facilitate pull-down of macrophage-expressed DUBs with affinity for WP1130 (Fig. 3). Activity-based DUB profiling utilizes an HA-tagged Ub (HA-Ub-vinyl sulfone; HA-UbVS), which irreversibly binds to the active site of DUBs [34]. RAW cells were treated with 5 µM WP1130 or DMSO prior to infection with MNV-1 or mock lysate, washed, and media containing WP1130 or DMSO added back for one hour. RAW cells were then lysed by sonication, and HA-tagged soluble proteins were detected by immunoblotting using an anti-HA antibody. Multiple DUBs reproducibly showed greater HA labeling upon infection with MNV-1 in DMSO-treated cells (Fig. 3A). In addition, some of these active DUBs were inhibited by WP1130 treatment following infection (Fig. 3A). Of particular interest was a band with the approximate molecular weight for USP14, a cellular DUB previously identified as a target of WP1130 in lymphoma cells [34] (Fig. 3A, arrow head). To specifically address whether USP14 activity was inhibited by WP1130 treatment, we labeled DUBs in mock- and MNV-1-infected RAW cells that were treated with DMSO or WP1130. Active DUBs were labeled with HA-UbVS, immunoprecipitated with an anti-HA antibody and USP14 was detected by immunoblot (Fig. 3B top). Four independent experiments demonstrated that WP1130 treatment significantly reduced USP14 activity in both mock- and virally-infected samples compared to DMSO, but not compared to each other (Fig. 3B, quantitation). As a control, total USP14 levels were examined by immunoblots in parallel experiments. No change in total USP14 protein levels were observed (Fig. 3B, middle), suggesting WP1130 did not cause USP14 degradation. Taken together, these results demonstrated that USP14 activity is inhibited by WP1130 in RAW cells without affecting total protein levels, and that USP14 activity and WP1130 inhibition of this activity is independent of viral infection.

To identify proteins that interacted with WP1130, we used a biotinylated version of WP1130 and its inactive analog (Fig. 1B) for pull-down experiments. No significant difference between the biotinylated and non-biotinylated WP1130 was detected in their ability to inhibit MNV-1 infection, while a chemically inactive, biotinylated version of WP1130 (Null-Biotin) did not reduce MNV-1 infection (Fig. 3C). This demonstrated that biotinylation of WP1130 did not affect its antiviral activity. Hence, uninfected RAW cells were incubated with either the biotinylated WP1130 (WP) or the inactive biotinylated analog (Null), lysed, and biotinylated proteins precipitated with streptavidin agarose beads (Invitrogen). Proteins were resolved by SDS-Page and stained with Sypro Ruby Red (Invitrogen) (Fig. 3D). Biotinylated WP1130, but not the inactive analog, pulled down a band of similar molecular weight (Fig. 3D, asterisk) as identified by the activity-based DUB profiling (see Fig. 3A). Trypsin-derived peptides from both lanes of this region in the gel were subjected to mass spectrometric analysis, which identified USP14 only in samples treated with biotinylated WP1130 but not the inactive analog. A total of five unique peptides were recovered from two independent experiments (Fig. S2, Table S1). In addition, five other proteins with at least two unique peptides were identified and found to be associated with the biotinylated but not the inactive analog of WP1130 in these two independent experiments (Table S1).

In summary, these results demonstrated that WP1130 binds and inhibits USP14 in murine macrophages independent of virus infection.

### Inhibition or knockdown of USP14 reduces MNV-1 non-structural gene expression

**Figure 4:**
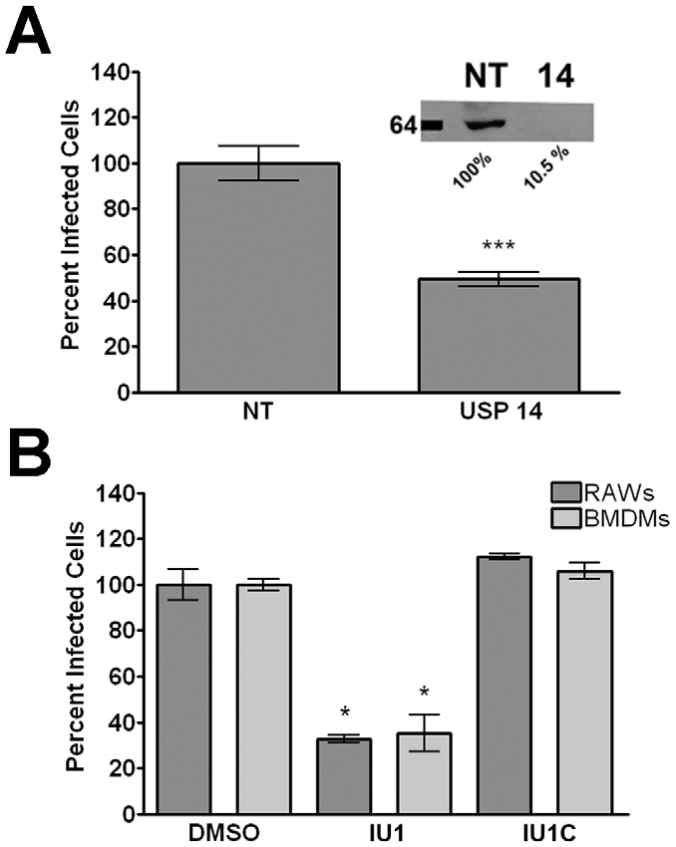
USP14 is required for optimal MNV-1 non-structural gene expression in murine macrophages. (A) siRNA knockdown of USP14 significantly reduces the number of MNV-1-infected RAW cells. Cells were transfected with non-targeting (NT) or USP14-targeted (USP14) Accell siRNA and infected with MNV-1 (MOI 5). Twelve hours post-infection, cells were fixed and stained with an anti-VPg antibody to quantify the number of infected cells. A representative immunoblot verifying protein knockdown in transfected cell lysates using an anti-USP14 antibody is also shown (inset). (B) The USP14 specific inhibitor IU1 decreases the number of virally infected murine macrophages. RAW cells and BMDMs were treated with the USP14 inhibitor IU1 or the inactive analog IU1C for 30 min prior to infection, and the number of MNV-1 infected cells quantitated 12 hours later by immunofluorescence as in (A). In all cases, data from three independent experiments with two experimental replicates per condition are presented as means +/− S.E.M. *P<0.05, *** *P*<0.001.

To elucidate the role of USP14 during MNV-1 infection, we used both protein knockdown and pharmacologic inhibition of USP14. First, RAW cells were transfected with siRNA targeting USP14 or a non-targeting (NT) control siRNA as previously described [41]. USP14 knockdown was verified by Western blot loading the same cell equivalents in each lane. USP14 protein levels were reduced to 20.5%+/−23.1% compared to the NT-treated RAW cells (Fig. 4A, inset). Transfected RAW cells were then infected with MNV-1 and the number of virally infected cells was determined by immunofluorescence staining for the MNV-1 non-structural gene VPg as previously described [41]. Following USP14 knockdown, MNV-1 VPg expression was significantly reduced by approximately 50% compared to the NT control (Fig. 4A), suggesting USP14 is required for MNV-1 non-structural gene expression. Although decreased viral titers following USP14 knockdown were also observed in RAW cells 8 hours post-infection by plaque assay, this was not statistically significant (data not shown).

To verify these results, we used an inhibitor of USP14, called IU1 [43], and tested its effects on MNV-1 infection (Fig. 4B). RAW cells or BMDMs were treated with 5 µM IU1 or IU1C, an inactive analog of IU1, prior to infection with MNV-1. The number of virally infected cells was determined as described above. IU1 and IU1C treatment did not significantly affect cell viability as measured by mitochondrial dehydrogenase activity using WST-1 (ROCHE) (Fig. S1). Similar to the USP14 siRNA knockdown studies, the number of MNV-1 infected cells significantly decreased following treatment with IU1, but not the inactive analog IU1C (Fig. 4B). However, no statistically significant differences were observed when viral titers were measured by plaque assay (data not shown). Such differences in the experimental outcome when measuring infected cells by immunofluorescence vs. infectious virus particles by plaque assay have been previously observed by our laboratory [40]. Unfortunately, we were unable to determine the requirements of USP14 for Norwalk virus replication due to excessive cell toxicity in replicon-containing HG23 cells after a 24 hour treatment with IU1 (data not shown).

Overall, our results indicated a requirement of USP14 for efficient MNV-1 non-structural gene expression. Furthermore, the difference in the antiviral effect of the specific USP14 inhibitor (Fig. 4B) compared with the antiviral effect of WP1130 (a broader spectrum DUB inhibitor) (see Fig. 2A, B) suggested that additional WP1130-targeted DUBs also promote MNV-1 infection.

### WP1130 does not inhibit proteasome activity

**Figure 5:**
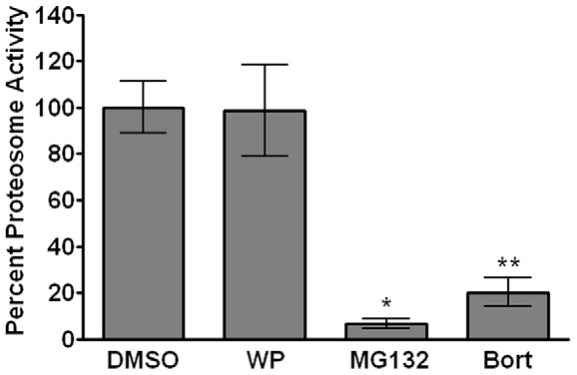
WP1130 treatment does not inhibit proteasome activity. RAW cells were treated with 5 µM WP1130 (WP), 50 µM MG132, 200 nM Bortezomib (Bort), or DMSO for 2 hours at 37°C. Equal amounts of protein from each cell lysate were incubated with 100 nM of the fluorogenic substrate Suc-LLVY-AMC for 60 minutes at 37°C. The fluorescence intensity for each sample was measured and normalized to the DMSO control. Data from three independent experiments with two experimental replicates per condition are presented as means +/− S.E.M. *P<0.05, ** *P*<0.01.

USP14 regulates proteasome activity and pharmacologic inhibition of USP14 increases the rate of protein degradation in the cell [43]. Previous reports demonstrated that WP1130 did not inhibit 20S proteasome activity *in vitro* and in lymphoma and leukemia cells, including the chymotrypsin-, trypsin- and caspase-like activities [34], [37]. To confirm that WP1120 did not affect proteasome activity in murine macrophages, we tested for chymotrypsin-like activity in RAW cells treated with WP1130, using two known proteasome inhibitors MG132 and Bortezomib [44], [45], or DMSO control as described [34] (Fig. 5). As anticipated the proteasome inhibitors MG132 and Bortezomib significantly reduced proteasome activity, while WP1130 treatment did not (Fig. 5). These results confirmed that WP1130's antiviral activity is not associated with 20S proteasome inhibition and suggested that DUBs play a critical role in other cellular processes important for MNV-1 infection.

### Activation of the unfolded protein response inhibits MNV-1 infection

**Figure 6:**
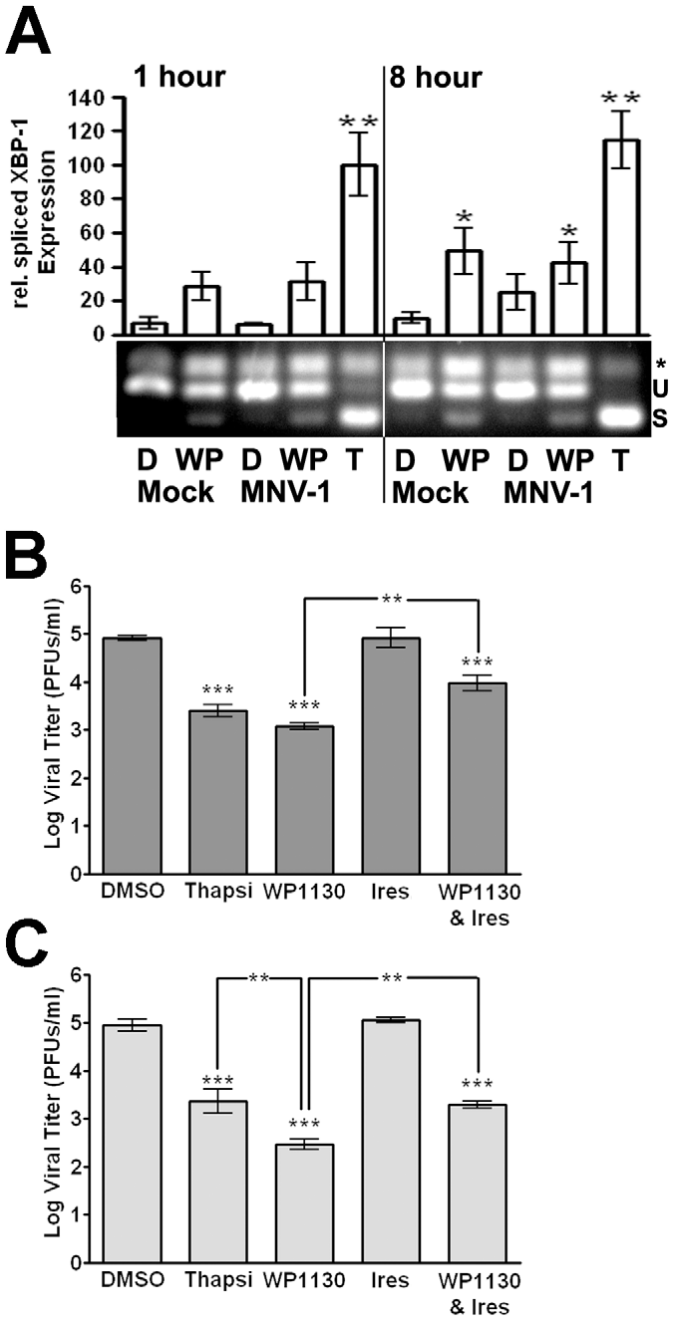
Activation of the UPR inhibits MNV-1 infection. (A) WP1130 treatment activates XBP-1. RAW cells were treated with 5 µM WP1130 (WP), 3 µM thapsigargin (T), or DMSO (D) and then mock- or MNV-1 infected (MOI 10). At 1 and 8 hours post-infection, RNA was isolated and XBP-1 message amplified. Activation of XBP-1 results in a faster migrating spliced form (s) of the unspliced XBP-1 (u). As previously observed [47], a hybrid PCR product was also detected (*). Densitometry was performed on three independent experiments, quantitated, and normalized to the 1 hr thapsigargin-treated sample. (B) UPR induction by thapsigargin inhibits MNV-1 infection in RAW cells, while the antiviral activity of WP1130 is partially rescued by Irestatin, an inhibitor of the IRE1 endonuclease activity. RAW cells were treated with DMSO, 3 µM thapsigargin (Thapsi), 5 µM WP1130, 2.5 µM Irestatin (Ires.), or both 2.5 µM Irestatin and 5 µM WP1130 (WP1130 & Ires.) for 30 min prior to MNV-1 infection (MOI 5). Viral titers were determined by plaque assay at 8 hours after infection. (C) UPR induction by thapsigargin inhibits MNV-1 infection in bone marrow-derived macrophages (BMDMs), while the antiviral activity of WP1130 is partially rescued by Irestatin. The experiment was carried out as described under (B), except MNV-1 titers were determined at 12 hours postinfection. In all cases, data from three independent experiments are presented as means +/− S.E.M. **P<0.01, and *** *P*<0.001.

In addition to regulating proteasome function, USP14 regulates the UPR by associating with inactive IRE1, although the mechanism of this regulation has not been elucidated [32]. We hypothesized that inhibition of USP14 by WP1130 would result in IRE1 activation, one of the three sensors of the UPR [22]. The active endonuclease domain of IRE1 splices the mRNA encoding XBP-1, which leads to expression of the active XBP-1 transcription factor [46]. To determine the effect of WP1130 treatment on IRE1, we measured splicing of XBP-1 mRNA in RAW cells treated with WP1130 or DMSO and then infected with MNV-1 or mock lysate (Fig. 6A). RNA was harvested at 1 and 8 hours post-infection. As a control, RAW cells were treated with 3 µM thapsigargin for 30 minutes to chemically induce the UPR. PCR was performed after cDNA synthesis using XBP-1 specific primers as previously described [47]. Three bands appeared upon XBP-1 amplification. Based on similar reports in the literature (reviewed in [48]), the top band is a hybrid PCR product (Fig. 6A, asterisk), while the middle and lower bands correspond to the unspliced (U) inactive and spliced (S) active forms of XBP-1, respectively (Fig. 6A). The intensity of the spliced XBP-1 product was also quantified across three independent experiments (Fig. 6A). WP1130 treatment induced XBP-1 activation as early as 1 hour after infection with MNV-1 or mock lysate and reached statistical significance at 8 hours post-infection, although not as robustly as the positive control thapsigargin (Fig. 6A). No difference in XBP-1 activation was observed between mock-infected and MNV-1-infected RAW cells at 1 hour post-infection. However, at 8 hours post-infection there was a faint and reproducible XBP-1 signal in MNV-1 infected cells, albeit not significantly different from DMSO controls. This suggested that MNV-1 infection may activate the UPR at later stages of the infectious cycle. Together our findings demonstrated that WP1130 treatment results in XBP-1 activation irrespective of MNV-1 infection.

To determine whether activation of the other two arms of the UPR, specifically PERK and ATF6, also occurred under the same conditions, immunoblots were performed using a phospho-specific PERK antibody and an antibody against ATF6 (Fig. S3). Phosphorylation of PERK was not observed after MNV-1 infection or WP1130 treatment at 1 or 8 hours post-infection, but was seen following thapsigargin treatment. Total PERK levels remained relatively stable across all conditions. No cleavage of inactivate ATF6 (ATF6 p90) into the active subunit (ATF6 p50) was observed during WP1130 treatment or MNV-1 infection, while WP1130 treatment followed by an 8 hour MNV-1 infection caused slight activation of ATF6. Robust activation of ATF6 was seen after thapsigargin treatment. While these results due not rule out the possibility of transient activation of PERK or ATF6, they suggest WP1130 activates the IRE1- but not PERK- or ATF6-dependent arms of the UPR.

Since UPR activation can inhibit viral infections [49], we investigated the effect of UPR activation on MNV-1 infection. RAW cells (Fig. 6B) or BMDMs (Fig. 6C) were treated with thapsigargin prior to infection and viral titers determined by plaque assay. MNV-1 titers were significantly reduced in murine macrophages treated with thapsigargin (Fig. 6B, C). This reduction was not significantly different to the antiviral effect observed with WP1130 treatment in RAW cells, while in BMDMs WP1130 treatment further inhibited MNV-1 infection. Since the IRE1-dependent arm of the UPR was induced upon WP1130 treatment, we tested whether inhibition of IRE1 with Irestatin, a specific inhibitor of the IRE1 endonuclease activity [50], could rescue the WP1130-induced block in MNV-1 infection. As a control, we first determined XBP-1 activation in RAW cells treated with 3 µM thapsigargin, 2.5 µM irestatin, and a combination of both inhibitors, or 2.5 µM irestatin, 5 µM WP1130, and irestatin and WP1130 combined. Irestatin inhibited most or all of the XBP-1 activation induced by thapsigargin or WP1130, respectively (Fig. S4). Next, murine macrophages were pre-treated with WP1130 or Irestatin alone or in combination and MNV-1 titers measured by plaque assay (Fig. 6B, C). RAW cells and BMDMs treated with both compounds produced significantly (∼50%) more viral progeny than WP1130 alone, while Irestatin treatment alone had no significant effect on MNV-1 titers. These results demonstrated that Irestatin can partly inhibit the antiviral effect of WP1130 to allow limited rescue of MNV-1 infection, suggesting that the anti-MNV-1 activity of WP1130 is in part mediated by IRE1. Interestingly, the anti-MNV-1 activity of thapsigargin was completely reversed by irestatin treatment (Fig. S5), suggesting that thapsigargin's antiviral effects are dependent on IRE1 endonuclease activity.

Taken together, these data showed that WP1130 treatment activated XBP-1 and that the IRE1 endonuclease activity was partly responsible for the anti-MNV-1 activity of WP1130, while the full antiviral effect of WP1130 is augmented by other non-IRE1-dependent mechanisms. Furthermore, pharmacologic activation of the UPR significantly inhibited MNV-1 infection in primary and cultured murine macrophages, identifying new targets for the development of anti-norovirus therapies.

### Activation of the UPR has broad antiviral effects

**Figure 7:**
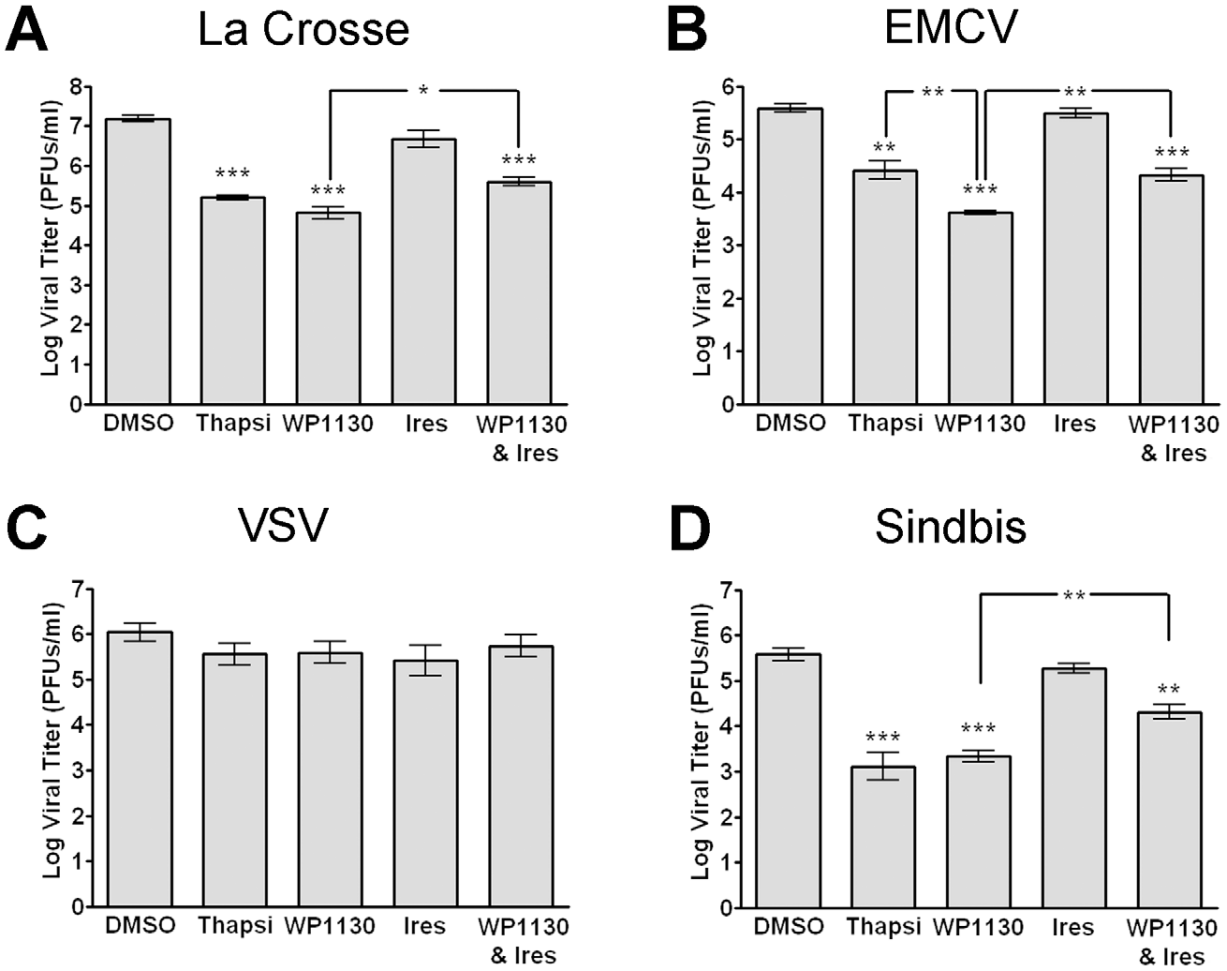
Activation of the UPR and WP1130 treatment show broad antiviral effects. (A–D) Cells were treated with DMSO, 3 µM thapsigargin (Thapsi), 5 µM WP1130, 2.5 µM Irestatin (Ires.), or both 2.5 µM Irestatin and 5 µM WP1130 (WP1130 & Ires.) prior to infection. (A) La Crosse virus infection of Be2-c cells is inhibited by WP1130 or thapsigargin. Treated Be2-c cells were infected with La Crosse virus (MOI 5) for 12 hours and viral titers determined by plaque assay on Vero cells. (B) Encephalomyocarditis virus (EMCV) infection of Vero cells is inhibited by WP1130 or thapsigargin. Treated Vero cells were infected with EMCV virus (MOI 5) for 12 hours and viral titers determined by plaque assay on Vero cells. (C) Vesicular stomatitis virus (VSV) infection of Vero cells is not inhibited by WP1130 or thapsigargin. Treated Vero cells were infected with VSV virus (MOI 5) for 12 hours and viral titers determined by plaque assay on Vero cells. (D) Sindbis virus infection of Vero cells is inhibited by WP1130 or thapsigargin. Treated Vero cells were infected with Sindbis virus (MOI 5) for 12 hours, and viral titers determined by plaque assay on Vero cells. In all cases, data from at least three independent experiments with two experimental replicates per condition are presented as means +/− S.E.M. *P<0.05, **P<0.01, and *** *P*<0.001.

Targeting host-specific functions and pathways such as the UPR may have broad-spectrum antiviral efficacy. Thus, we tested the antiviral effect of WP1130 and induction of the UPR on additional viruses with positive- and negative-sense RNA genomes and enveloped or non-enveloped virus particles. Be2-c cells (Fig. 7A) or Vero cells (Fig. 7B–D) were treated with thapsigargin, WP1130, Irestatin, WP1130 and Irestatin, or DMSO prior to infection. Cells were then infected with La Crosse virus, an enveloped negative-strand RNA virus (Fig. 7A), EMCV, a non-enveloped positive-strand RNA virus (Fig. 7B), VSV, an enveloped negative-strand RNA virus (Fig. 7C), or Sindbis virus, an enveloped positive-strand RNA virus (Fig. 7D). Both WP1130 and thapsigargin treatment significantly reduced La Crosse virus, EMCV, and Sindbis virus but not VSV progeny production, suggesting that activation of the UPR through thapsigargin can inhibit certain virus infections. Similar to findings with MNV-1, cells treated with Irestatin and WP1130, but not Irestatin alone, showed a small (∼50%) but significant rescue of La Crosse virus, EMCV, and Sindbis virus infections compared to WP1130 treatment alone (Fig. 7A, B, D). We did not observe a significant inhibition of infection with any of the treatments during VSV infection (Fig. 7C). Taken together, these data demonstrate that UPR activation is inhibitory to many but not all RNA viruses, and that the antiviral activity of WP1130 is mediated in part by the IRE1-dependent arm of the UPR.

### WP1130 inhibits MNV-1 infection of mice

**Figure 8:**
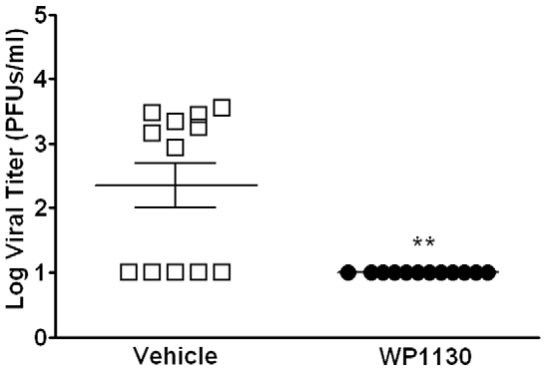
WP1130 inhibits MNV-1 infection in mice. Balb/c mice were administered 30 mg/kg of WP1130 dissolved in 20% DMSO and 80% PEG200 or vehicle control once daily via oral gavage. Mice were infected orally with 1×10^6^ PFUs of MNV-1 four hours after the first dose of WP1130. After 72 hours of infection, tissues were harvested along the gastrointestinal tract and viral titers determined by plaque assay. Shown are viral titers in the jejunum/duodenum of mice treated with WP1130 (empty box) or vehicle control (filled circle). Each symbol represents one animal. Data are from three independent experiments and are presented as means +/− S.E.M. **P<0.01.

To test the effectiveness of WP1130 in a mouse model, Balb/c mice were administered 30 mg/kg WP1130 dissolved in 20% DMSO and 80% PEG200 or vehicle control daily by oral gavage. Mice were orally infected four hours after the first WP1130 administration with 1×10^6^ PFUs of MNV-1. Three days post-infection, mice were harvested and viral titers in the gastrointestinal tract determined by plaque assay. A significant decrease in viral titers was observed in the jejunum/duodenum, the most proximal part of the gastrointestinal tract, in mice treated with WP1130 compared to vehicle control treated mice (Fig. 8). However, no significant differences were observed in more distal regions of the gastrointestinal tract. The limited effectiveness of WP1130 against MNV-1 in a region closest to the site of administration is most likely due to low solubility, experimentally determined to be 1.2 µg/ml, and/or poor bioavailability. These data suggest that WP1130 also possesses anti-MNV activity *in vivo* but further modifications of WP1130 are needed to increase its solubility and pharmacokinetic properties.

## Discussion

The functions of DUBs required during virus replication are poorly understood, and there are currently no DUBs reported to regulate norovirus replication. Using a small molecule inhibitor of a subset of cellular DUBs, WP1130, we demonstrated that MNV requires some DUBs during viral replication in macrophages. Specifically, USP14 was identified as a direct target of WP1130 in murine macrophages. Of the two known functions of USP14, *i.e.* regulation of proteasomal degradation or modulation of the UPR, changes in proteasome activity were not detected during WP1130 treatment. Instead, activation of the UPR as indicated by XBP-1 splicing was induced by WP1130. The antiviral activity of WP1130 was in part mediated by the UPR sensor IRE1 as treatment with Irestatin, a specific inhibitor of the IRE-1 endonuclease activity, partially rescued MNV-1 infection in the presence of WP1130. Similar findings were made with the other RNA viruses La Crosse virus, EMCV, and Sindbis virus. In addition, activation of the UPR with thapsigargin, a widely used UPR activator, also exhibited broad spectrum antiviral activity. These data are consistent with a model whereby induction of the UPR through inhibition of cellular DUBs blocks viral infection.

The activity of cellular DUBs during norovirus infection has not been addressed previously. Our work demonstrates for the first time that a cellular DUB, the proteasome-associated USP14, is required for optimal MNV-1 infection of murine macrophages. The mechanism by which USP14 inhibits MNV-1 infection remains to be defined. We hypothesize that one mechanism involves its interaction with IRE1 and activation of downstream UPR targets. Alternatively, USP14 interactions with viral or host proteins essential during norovirus infection may also play a role. Additional DUBs remain to be identified as antiviral effectors since specific inhibition or knockdown of USP14 was unable to recapitulate the entire antiviral activity of WP1130 (see Fig. 2 and 4). To date, we have tested two previously identified targets of WP1130, USP5 and USP9x [34], using siRNA knockdown. However, no changes in MNV-1 titers were observed (data not shown). This suggested only some of the DUBs targeted by WP1130 exhibit antiviral activity, enabling the development of more specific small molecule DUB inhibitors with anti-norovirus activity.

Our work also demonstrates that induction of the UPR with thapsigargin or WP1130 inhibits MNV-1 infection in murine macrophages (see Fig. 6). The antiviral effect of WP1130 was partially reversed by inhibition of the IRE1 endonuclease activity through Irestatin. Interestingly though, the other two arms of the UPR response, PERK and ATF6, were not activated by WP1130 treatment or MNV-1 infection (see S3). This suggests that the IRE1/XBP-1 arm of the UPR is sufficient to limit MNV-1 infection. The downstream effectors of the UPR that mediate viral inhibition remain to be defined. One attractive hypothesis is the link between the UPR and lipid metabolism, whereby ER stress results in the XBP-1-dependent activation of phospholipid biosynthesis pathways [51]. The recruitment of host membranes to viral replication sites or virus factories is a common requirement for positive-strand RNA viruses, such as MNV-1 and EMCV [52]. Therefore, we speculate that activation of the UPR prior to MNV-1 infection might limit the amount of membrane available for the virus to recruit to its replication sites. Interestingly, a slight activation of XBP-1 splicing is observed later during MNV-1 infection. This suggests that timing of UPR induction may be critical during infection, whereby UPR induction prior to or early during MNV-1 infection inhibits infection, while UPR induction late in the viral life cycle has no effect or promotes infection. Indeed, WP1130 post-treatment of murine macrophages does not inhibit MNV-1 infection (see Fig. 2). This is similar to findings with West Nile virus, strain Kunjun, which is also sensitive to thapsigargin treatment early but not late in infection [53]. Interestingly, inhibition of Norwalk virus replication by WP1130 was not as strong as MNV-1 replication (see Fig. 2). This may suggest differences in the ability of both viruses to directly modulate the UPR. Another explanation may be that in the replicon system the virus has already established replication factories and may need less membrane synthesis. It is further conceivable that inhibition of membrane synthesis may not be the only mechanism by which WP1130 inhibits norovirus infections and that transient signaling from other branches of the UPR, or signaling through other IRE1 adaptors, such as JNK [54] play a role. Further characterizing the role of the UPR during norovirus infection could lead to novel insights into norovirus biology and identification of new antiviral drug targets.

In addition to MNV-1, induction of the UPR also had antiviral effects against the RNA viruses ECMV, La Crosse virus, and Sindbis virus, but not VSV (see Fig. 7). Protein synthesis during viral infection in general is thought to induce the UPR, and experimental evidence demonstrates Sindbis virus and VSV infections induce ER stress [55], [56]. However, the consequences of UPR induction for individual virus infections are less well understood. For other RNA viruses such as HCV or rotavirus, induction of the UPR can lead to inhibition of viral infection, while modulation of the UPR by viral proteins can facilitate infection [57], [58], [59], [60], [61], [62]. For HCV, the IRE1/XBP-1 arm of the UPR is suppressed in replicon-containing cells resulting in decreased ER-associated protein degradation [57]. Since WP1130 inhibits viral infection in part in an IRE1-dependent manner, active suppression of this pathway by HCV would make HCV insensitive to WP1130. Consistent with that hypothesis is our observation that WP1130 did not inhibit HCV replication in HCV replicon-containing cells (data not shown). We speculate that viruses would only be sensitive to WP1130 treatment if activation of the IRE1/XBP-1 arm of the UPR inhibits viral replication.

The mechanism by which UPR activation or DUB inhibition could inhibit enveloped virus infection remains unclear. Induction of the degradative capacity of the UPR could reduce the stability of viral glycoproteins that are required for virion formation, since these proteins traffic through the ER. Degradation of HCV glycoproteins via the UPR has been observed in infected cells [58]. Other mechanisms of inhibition may be mediated through modulation of membrane synthesis [51], since virus budding and viral replication factories rely on cellular membranes. Investigations into the role of the UPR and DUBs during Sindbis and La Crosse virus infections will help elucidate these cell-intrinsic antiviral mechanisms.

Last but not least, our work also demonstrated that WP1130 treatment inhibits MNV-1 infection in a mouse model. While this inhibition was limited to the closest site of administration, the jejunum/duodenum, we hypothesize that the solubility and/or bioavailability of WP1130 are the main reason behind this spatially restricted antiviral effect. Thus, these findings support the notion that the WP1130 chemical scaffold provides a good starting point for future drug development efforts.

In summary, we demonstrate that blocking DUB activities and induction of UPR inhibits infection of the non-enveloped viruses MNV-1 and EMCV, and the enveloped viruses Sindbis virus and La Crosse virus. Therefore, targeting DUBs and/or the UPR with small molecules may provide a unique pathway for broad spectrum antiviral therapies.

## Materials and Methods

### Cell culture and mice

RAW 264.7 cells were purchased from ATCC (Manassas, VA) and maintained as previously described [14]. Swiss Webster and Balb/c mice were purchased from Charles River and housed at the University of Michigan in accordance with all federal and university policies as outlined by the Guide for the Care and Use of Laboratory Animals and approved by the University of Michigan Committee on Use and Care of Animals. Bone marrow-derived macrophages (BMDMs) were isolated as previously described [14]. HG32 cells containing the Norwalk virus replicon were cultured as previously described [10]. Vero cells and Be2-(c) cells were purchased from ATCC (Manassas, VA) and maintained as suggested by ATCC.

### Virus stocks

The plaque purified MNV-1 clone (GV/MNV1/2002/USA) MNV-1.CW3 [63] was used at passage 6 for all experiments. Encephalomyocarditis virus, Sindbis virus, and La Crosse virus were obtained from Dr. David Miller (University of Michigan) and propagated as previously described [64].

### Small molecule inhibitors

All small molecules were dissolved in DMSO, except ribavirin (dissolved in PBS). WP1130, biotinylated WP1130, biotinylated WP1130 null probe, and the inactive USP14 inhibitor IU1C were synthesized by the Vahlteich Medicinal Chemistry Core (University of Michigan). Ribavirin, MG132, Bortezomib, and thapsigargin were obtained from Sigma-Aldrich. The USP14 inhibitor IU1 was obtained from OTAVA LTD. Irestatin was purchased from Axon Medchem.

### Growth curves

RAW cells, BMDMs, Be-2c, or Vero cells were plated at 2×10^5^ cells/ml in 12-well plates and allowed to attach overnight. Cells were then incubated with the concentrations of inhibitors and lengths of time as indicated. Next, cells were infected with an MOI of 5 with the indicated virus for one hour on ice. Infected cells were washed three times with ice-cold PBS. Media containing the appropriate inhibitors was added back to cells and the infection was allowed to proceed until the indicated time point. The cells were freeze-thawed twice, and viral titers were determined by plaque assay as previously described on RAW cells for MNV-1 or on Vero cells for all other viruses [14].

### Immunofluorescence assay

RAW cells or BMDMs were plated at 2×10^5^ cells/ml in 6-well plates containing sterile glass coverslips (Fisher Scientific) and allowed to attach overnight. Cells were then infected as described above. Infection was allowed to proceed until the indicated time point when the cells were fixed with 4% paraformaldehyde in PBS for ten minutes, washed once with PBS, and stained for the viral non-structural protein VPg [65] as previously described [66].

### Neutral red assay

RAW cells were plated at 1×10^6^ cells/ml in 6-well plates and allowed to attach overnight. For pre-treatments, cells were incubated with 5 µM WP1130 or DMSO for 30 min. Cells were infected with MNV-1 at an MOI of 0.001 in the presence of 5 µM WP1130, or DMSO. After 60 min, the infection was exposed to white light and a plaque assay preformed as previously described [41]. To assess the non-specific effects of the compound (*i.e.* post-treatment), cells were infected for 60 minutes at an MOI of 0.001 in the absence of inhibitor, virus particles not yet uncoated were inactivated by exposure to white light, and inhibitor was added back for an equal length of time as the pre-treatment. To determine the dynamic range of the experiment, DMSO treated cells were infected at an MOI of 0.001 and illuminated 0 minutes or 60 minutes after addition of virus.

### Binding assay

RAW cells were plated at 2×10^5^ cells/ml in 12-well plate and allowed to attach overnight. The following day, cells were treated with 5 µM WP1130 or DMSO (control) for 30 minutes. The cells were then placed on ice and media aspirated. Media containing MNV-1 at an MOI of 5 and DMSO or 5 µM WP1130, was added to the plate for 1 hour on ice. Cells were then washed three times with ice-cold PBS. After the final wash, RNA was isolated with Trizol (Invitrogen) following the manufacturer's recommendations. Viral cDNA was then prepared and genome titers measured by qRT-PCR as previously described [40].

### MNV-1 replication assay

4×10^5^ RAW cells were transfected with 1 µg of viral RNA obtained from TRIZOL extraction of viral lysate using 8 µl Lipofectamine 2000 (Invitrogen) for 6 hours in OPTIMEM media. RNA was harvested from transfected cells after 12 hours using Trizol (Invitrogen). In parallel, media from additional samples was aspirated and media containing DMSO or 5 µM WP1130 was added back to the cells for an additional 12 hours. RNA was harvested from cells and viral genomes were quantitated as previously described [40].

### HuNoV replicon assay

HG23 cells containing the Norwalk virus replicon plasmid under G418 selection were plated at 2×10^5^ cells in 6-well plates and allowed to attach overnight. After 24 hours media was aspirated, and DMSO or 5 µM WP1130 was added back. Cells were incubated for an additional 24 hours, at which time RNA was isolated from cells using TRIZOL (Invitrogen). Norwalk virus genomes were then quantitated by qRT-PCR as previously described [10].

### Streptavidin precipitation assay

RAW cells were plated at 1×10^7^ cells in T75 flasks and allowed to attach overnight. The following day, cells were treated with 5 µM biotinylated WP1130 or 5 µM of the biotinylated inactive analog of WP1130 for 30 minutes. The cells were then placed on ice and media aspirated. Media containing DMSO, 5 µM biotinylated WP1130 or 5 µM biotinylated inactive analog of WP1130 were added to the plate for 1 hour on ice. Cells were then washed three times in ice-cold PBS. After the final wash media containing the indicated inhibitor was added. The cells were incubated for an additional hour at 37°C, and then lysed in RIPA buffer on ice for ten minutes. Insoluble protein was removed by high-speed centrifugation at 16.1 RCF for 30 minutes at 4°C. Soluble protein was added to agarose beads conjugated to streptavidin (Invitrogen) and incubated with shaking at 4°C overnight. The next day, agarose beads were washed four times with PBS containing Complete Mini, EDTA-free protease tablets (Roche), boiled in 2× SDS PAGE sample buffer, and loaded onto SDS PAGE gels. Proteins were visualized using Sypro Ruby Red fluorescent protein stain (Invitrogen) according to the manufacturer's instructions. The indicated bands and corresponding region in the control lane were then cut and sent for mass spectrometry analysis at the University of Michigan Pathology Mass Spectrometry Lab.

### Deubiquitinase labeling assay

RAW cells were plated at 1×10^6^ cells/ml in 6-well plates and allowed to attach overnight. The following day cells were treated with 5 µM WP1130 or DMSO for 30 minutes at 37°C. Cells were then placed on ice, media aspirated, and replaced with media containing 5 µM WP1130 or DMSO. Next, cells were infected on ice with mock lysate, or MNV-1 at an MOI of 10 for 1 hour. Cells were washed three times with ice-cold PBS, and media containing WP1130 or DMSO was added back to the cells. Cells were then incubated for 1 hour at 37°C, placed on ice and washed once with ice-cold PBS, scraped into PBS and pelleted. DUB labeling buffer (50 mM Tris-HCl, pH 7.5, 0.5% NP-40, 5 mM MgCl_2_, 150 NaCl, and complete mini EDTA-free protease inhibitor cocktail (Roche) was added to the cell pellet. RAW cells were then sonicated for 3 seconds at a power of 3 using a Microson Ultrasonic Cell Disruptor XL with a microprobe tip (Misonix). Insoluble proteins were removed by centrifugation at 16.1 RCF for 30 minutes at 4°C. The concentration of the cell lysates was determined by Bradford assay. Protein samples (20 µg) were added to 200 ng of HA-Ubiquitin Vinyl Sulfone (Boston Biochem) and incubated at 37°C for 90 minutes. Next, samples were diluted in RIPA buffer on ice, and incubated with 2 µg of an anti-HA antibody (Invitrogen) for 1 hour on ice with gentle mixing. Protein A-coated agarose beads (Invitrogen) were added and incubated overnight with rocking at 4°C. The next day the agarose beads were washed four times with PBS containing Complete Mini, EDTA-free protease tablets (Roche), boiled in SDS Page sample buffer, and loaded onto SDS PAGE gels. After SDS PAGE, gels were transferred to nitrocellulose membrane and an immunoblot was performed using an anti-USP14 antibody (Abcam) at a dilution of 1∶2000 and a secondary goat anti-mouse HRP dilution of 1∶5000 as described below.

### Proteasome activity assay

RAW cells were plated at 1×10^6^ cells/ml in 6-well plates and incubated overnight. Cells were treated with 5 µM WP1130, 50 µM MG132, 200 nM Bortezimib, or DMSO for 2 hours at 37°C. Next, cells were placed on ice, washed with PBS, and lysed in ice-cold lysis buffer (50 mM HEPES [pH 7.5], 5 mM EDTA, 150 mM NaCl, and 1% Triton X-100). Insoluble protein was removed by centrifugation as described above, and protein concentrations were determined by Bradford assay. Each sample (10 µg) was added to 100 nM fluorogenic substrate, Suc-LLVY-AMC (Boston Biochem), which measures chymotrypsin activity, including 20S proteasome activity [34]. After incubation for 60 minutes at 37°C, the fluorescent intensity of each sample was determined using a Synergy HT plate reader (Bio Tek). Fluorescent intensities were normalized to DMSO control.

### XBP-1 RT-PCR

RAW cells were plated at 1×10^6^ cells/ml in 6-well plates and allowed to attach overnight. Next, cells were treated with 5 µM WP1130, 3 µM thapsigargin, or DMSO for 30 minutes at 37°C. Cells were then placed on ice, media aspirated, and media containing appropriate inhibitors with mock lysate, or MNV-1 at an MOI of 10 was added back for 1 hour. Cells were washed three times with ice-cold PBS and media containing appropriate inhibitors added back. At the times indicated, RNA was isolated using the SV Total RNA Isolation Kit (Promega), and cDNA was synthesized followed by PCR amplification using XBP-1 specific primers as previously described [48]. PCR products were run on a 3% agarose gel and visualized with SYBR Green (Invitrogen) on an Alpha Imager HP (Alpha Innotech). Band intensities were quantitated as previously described [41].

### siRNA knockdown

RAW cells were plated at a density of 2×10^5^ cells/ml in a 6-well plate and incubated overnight. Protein knockdown was performed as previously described [41]. Briefly, cells were washed with Accell siRNA Delivery Media (Dharmacon), and incubated with Accell siRNA delivery media containing 1 µM of the indicated siRNA. After 72 hours, RAW cells were washed once with DMEM and incubated in DMEM overnight. Cells were then infected as described above for 12 hours. RAW cells were analyzed by immunofluorescence assay or western blot as described herein.

### Western blot analysis

Whole cell lysates from 2×10^5^ RAW cells were generated by adding 2× SDS-PAGE sample buffer to cells and boiling samples for 5 minutes. Lysates were separated by SDS-PAGE, and immunoblots performed as previously described [41]. The following antibodies and dilutions were used: 1∶2000 anti-HA (Abcam, cat. no. ab18181), 1∶2000 anti-PERK (Cell Signaling, cat. no. 3192s), 1∶2000 anti-phospho PERK (Cell Signaling, cat. no. 3179s), 1∶2000 anti-ATF6 (Abcam, cat. no. ab11909), and 1∶2000 anti-USP14 (Abcam, cat. no. ab56210). Band densities were quantitated as previously described [41].

### WP1130 in vivo efficacy study

Twenty four 6–8 week old Balb/c mice were administered 30 mg/kg of WP1130 dissolved in 20% DMSO and 80% PEG200 or vehicle control via oral gavage using a 1.25 mm ball diameter gauge oral gavage needle (Cadence Science). Mice were allowed to recover for four hours, and then infected by placing 1×10^6^ PFUs of MNV-1 in a volume of 30 µl into the mouth with a micropipette. Mice were administered additional 30 mg/kg doses of WP1130 or vehicle control by oral gavage at 24 and 48 hours after infection. Tissues were harvested 72 hours after infection. Viral titers for various regions along the entire length of the gastrointestinal tract were determined by plaque assay as previously described [14].

### Statistics

Error bars represent standard error between at least three independent experiments with at least two replicates per condition. Statistical analysis was performed using the Prism Software v 5.01 (GraphPad Software). The two-tailed student t-test was used to determine statistical significance. * p<0.05, ** p<0.01, and *** p<0.001.

## Supporting Information

Figure S1 WP1130 does not affect cell viability. RAW cells (RAWs) or primary bone marrow-derived macrophages (BMDMs) were treated with DMSO, 5 µM WP1130, 5 µM IU1, or 5 µM IU1C for 30 min prior to incubation on ice for one hour, three washes with ice-cold PBS, and incubation at 37°C in the presence of the compound for 8 (in RAWs) or 12 (in BMDMs) hours. Cells were then washed once with PBS, and WST-1 reagent diluted 1 to 10 in media. OD_420_ was determined 90 minutes after addition of WST-1 and normalized to the DMSO treated cells.
(TIF)

Figure S2 USP14 sequence with identified peptides. The USP14 amino acid sequence is shown with the individual peptides identified by mass spectrometry highlighted in bold and underlined.
(TIF)

Figure S3 WP1130 treatment or MNV-1 infection do not activate PERK or ATF6 in RAW cells. RAW cells were treated with DMSO (D), 5 µM WP1130 (WP), or 3 µM thapsigargin (T) for 30 min prior to MNV-1 infection (MNV-1) or mock (Mock) infection for one hour on ice. Inoculums were washed off and media containing DMSO, 5 µM WP1130, or 3 µM thapsigargin added back to cells. Cells were lysed in SDS Page sample buffer 1 and 8 hours post-infection and separated on a 10% SDS-PAGE gel. Immunoblots were performed to determine phosho-PERK levels (pPERK), total PERK levels (PERK) or cleavage of ATF6 (ATF6 p90, ATF6 p50). Images are a representation of two experiments.
(TIF)

Figure S4 Irestatin inhibits XBP-1 splicing in RAW cells. RAW cells were treated for eight hours with 3 µM thapsigargin (Thapsi), 2.5 µM Irestatin (Ires), and both 2.5 µM Irestatin and 5 µM WP1130 (Ires & T), or 2.5 µM Irestatin (Ires), 5 µM WP1130 (WP1130), or both (Ires & WP). RNA was isolated and XBP-1 message amplified. Activation of XBP-1 results in a faster migrating spliced form (s) of the unspliced XBP-1 (u). As previously observed [47], a hybrid PCR product was also detected (*).
(TIF)

Figure S5 Irestatin inhibits thapsigargin's anti-MNV-1 effect in RAW cells. RAW cells were treated with DMSO (DMSO), 3 µM thapsigargin (Thapsi), 2.5 µM Irestatin (Ires), or a combination of both inhibitors (Ires & Thapsi) for 30 min prior to MNV-1 infection for one hour on ice. Inoculums were washed off with 3 washes of ice-cold PBS, and media containing inhibitors added back to cells for 8 hours. Viral titers were determined by plaque assay. Data from three independent experiments with two experimental replicates per condition are presented as means +/− S.E.M. *** *P*<0.001.
(TIF)

Table S1 List of identified proteins with at least two unique peptides associated with the biotinylated but not the inactive analog of WP1130.
(DOC)
