# Supplementary figures and images for: Antiviral Activity of a Small Molecule Deubiquitinase Inhibitor Occurs via Induction of the Unfolded Protein Response
Source: PLoS Pathog. 2012 Jul 5;8(7):e1002783. doi: 10.1371/journal.ppat.1002783 (PMC3390402; doi:10.1371/journal.ppat.1002783)

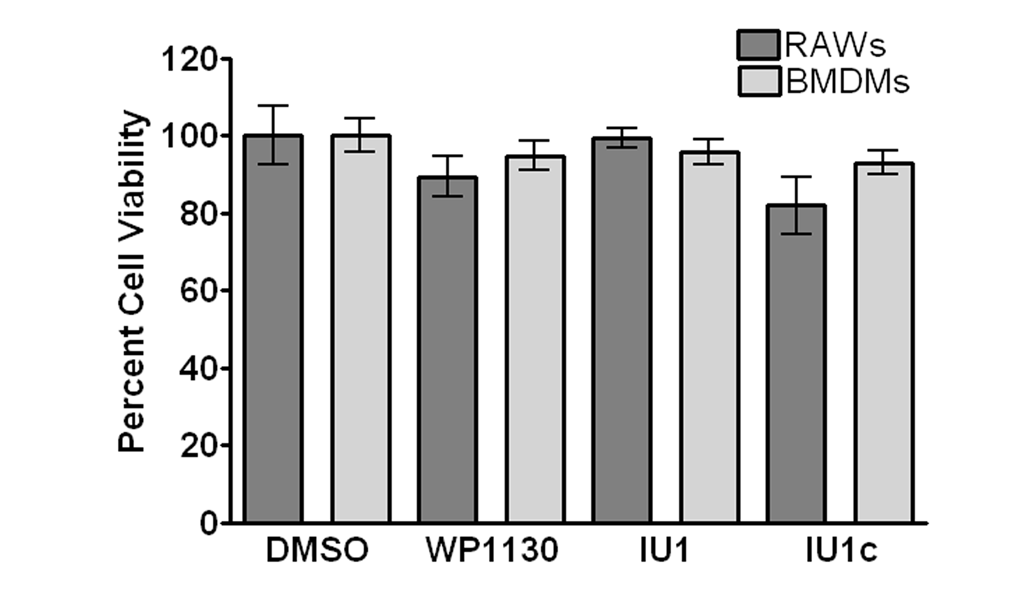

Supplement: Figure S1 — WP1130 does not affect cell viability. RAW cells (RAWs) or primary bone marrow-derived macrophages (BMDMs) were treated with DMSO, 5 µM WP1130, 5 µM IU1, or 5 µM IU1C for 30 min prior to incubation on ice for one hour, three washes with ice-cold PBS, and incubation at 37°C in the presence of the compound for 8 (in RAWs) or 12 (in BMDMs) hours. Cells were then washed once with PBS, and WST-1 reagent diluted 1 to 10 in media. OD420 was determined 90 minutes after addition of WST-1 and normalized to the DMSO treated cells. (TIF) [file ppat.1002783.s001.tif]

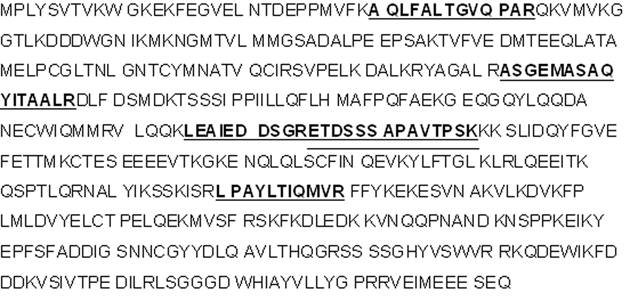

Supplement: Figure S2 — USP14 sequence with identified peptides. The USP14 amino acid sequence is shown with the individual peptides identified by mass spectrometry highlighted in bold and underlined. (TIF) [file ppat.1002783.s002.tif]

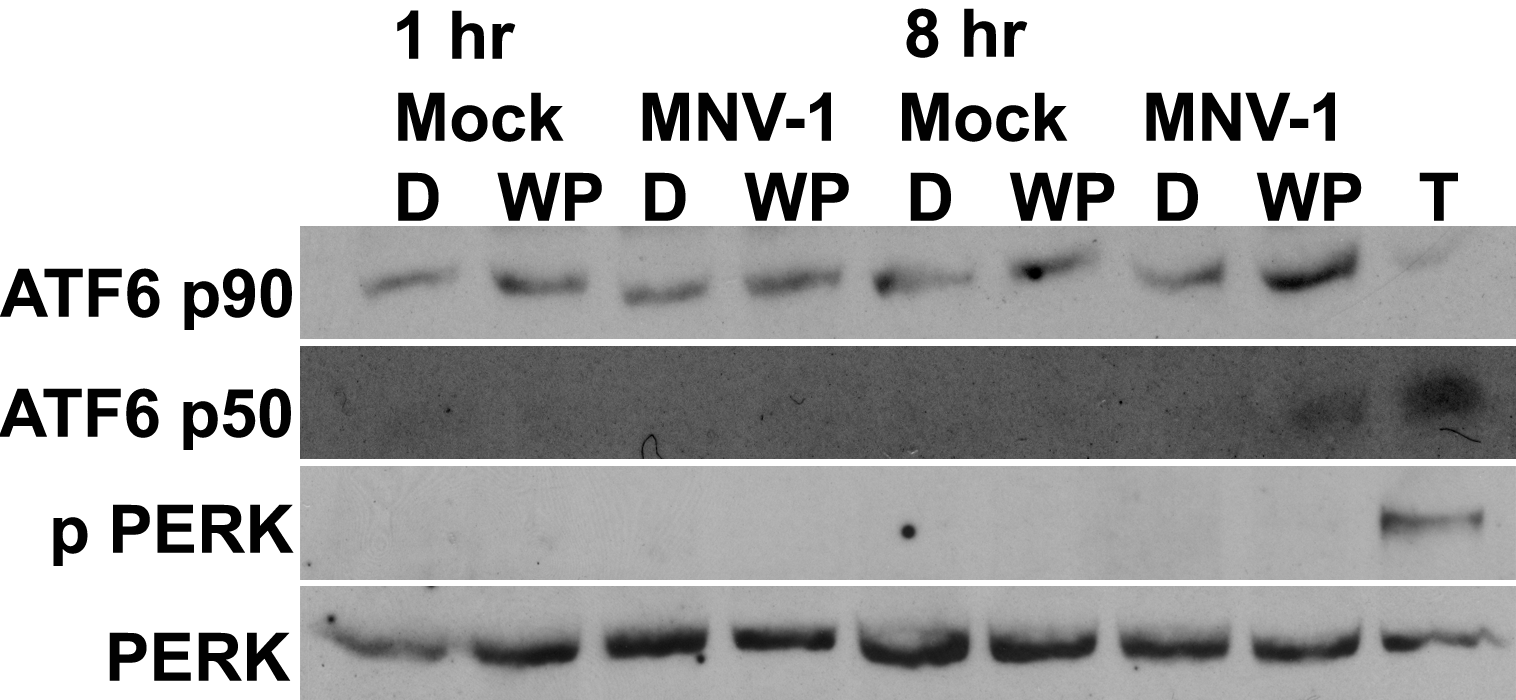

Supplement: Figure S3 — WP1130 treatment or MNV-1 infection do not activate PERK or ATF6 in RAW cells. RAW cells were treated with DMSO (D), 5 µM WP1130 (WP), or 3 µM thapsigargin (T) for 30 min prior to MNV-1 infection (MNV-1) or mock (Mock) infection for one hour on ice. Inoculums were washed off and media containing DMSO, 5 µM WP1130, or 3 µM thapsigargin added back to cells. Cells were lysed in SDS Page sample buffer 1 and 8 hours post-infection and separated on a 10% SDS-PAGE gel. Immunoblots were performed to determine phosho-PERK levels (pPERK), total PERK levels (PERK) or cleavage of ATF6 (ATF6 p90, ATF6 p50). Images are a representation of two experiments. (TIF) [file ppat.1002783.s003.tif]

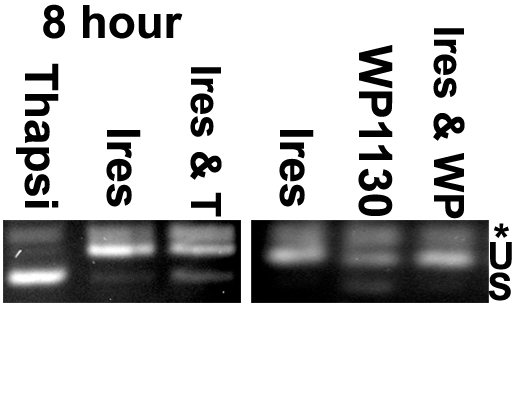

Supplement: Figure S4 — Irestatin inhibits XBP-1 splicing in RAW cells. RAW cells were treated for eight hours with 3 µM thapsigargin (Thapsi), 2.5 µM Irestatin (Ires), and both 2.5 µM Irestatin and 5 µM WP1130 (Ires & T), or 2.5 µM Irestatin (Ires), 5 µM WP1130 (WP1130), or both (Ires & WP). RNA was isolated and XBP-1 message amplified. Activation of XBP-1 results in a faster migrating spliced form (s) of the unspliced XBP-1 (u). As previously observed [47], a hybrid PCR product was also detected (*). (TIF) [file ppat.1002783.s004.tif]

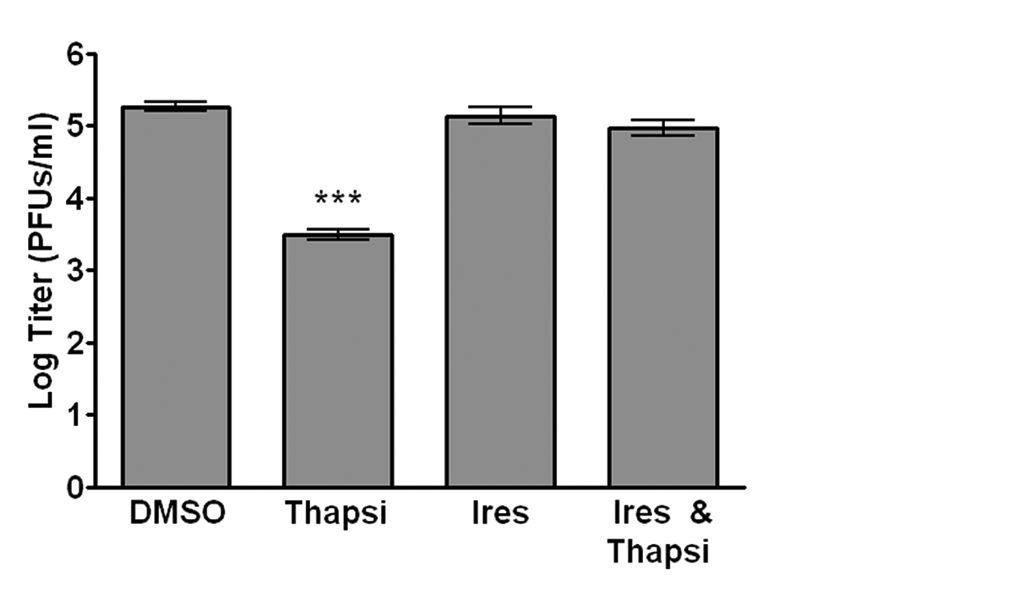

Supplement: Figure S5 — Irestatin inhibits thapsigargin's anti-MNV-1 effect in RAW cells. RAW cells were treated with DMSO (DMSO), 3 µM thapsigargin (Thapsi), 2.5 µM Irestatin (Ires), or a combination of both inhibitors (Ires & Thapsi) for 30 min prior to MNV-1 infection for one hour on ice. Inoculums were washed off with 3 washes of ice-cold PBS, and media containing inhibitors added back to cells for 8 hours. Viral titers were determined by plaque assay. Data from three independent experiments with two experimental replicates per condition are presented as means +/− S.E.M. *** P<0.001. (TIF) [file ppat.1002783.s005.tif]
