# Supplementary material for: Antiviral Activity of a Small Molecule Deubiquitinase Inhibitor Occurs via Induction of the Unfolded Protein Response
Source: PLoS Pathog. 2012 Jul 5;8(7):e1002783. doi: 10.1371/journal.ppat.1002783 (PMC3390402; doi:10.1371/journal.ppat.1002783)
Supplement: Table S1 — List of identified proteins with at least two unique peptides associated with the biotinylated but not the inactive analog of WP1130. (DOC) [file ppat.1002783.s006.doc]

**Table S1** List of identified proteins with at least two unique peptides associated with the biotinylated but not the inactive analog of WP1130

Name (Gene Symbol) # of identified peptides *

| Ubiquitin Specific Protease 14 (USP14) | **2, 3** |
| --- | --- |
| Protein Phosphatase 2 (PP2) | **6, 14** |
| Heat Shock Protein 1 (Hspd 1) | **4, 20** |
| Chaperonin containing Tcp1, subunit 3 (Cct3) | **8, 4** |
| Lamin A (Lmna) | **4, 13** |
| Chaperonin containing Tcp1, subunit 2 (Cct2) | **2, 10** |

* number of peptides identified from independent experiments separated by a comma.
